# Improved limit of detection for zoonotic *Plasmodium knowlesi* and *P. cynomolgi* surveillance using reverse transcription for total nucleic acid preserved samples or dried blood spots

**DOI:** 10.1101/2024.04.04.24305339

**Published:** 2024-04-06

**Authors:** Kamil A Braima, Kim A Piera, Inke ND Lubis, Rintis Noviyanti, Giri S Rajahram, Pinkan Kariodimedjo, Irbah RA Nainggolan, Ranti Permatasari, Leily Trianty, Ristya Amalia, Sitti Saimah binti Sakam, Angelica F Tan, Timothy William, Jacob AF Westaway, PingChin Lee, Sylvia Daim, Henry Surendra, Nathaniel Christy, Andrew G Letizia, Christopher L Peatey, Mohd Arshil Moideen, Bridget E Barber, Colin J Sutherland, Nicholas M Anstey, Matthew J Grigg

**Affiliations:** 1. Global and Tropical Health Division, Menzies School of Health Research and Charles Darwin University, Darwin, Northern Territory, Australia; 2. Faculty of Medicine, Universitas Sumatera Utara, Medan, Sumatera Utara, Indonesia; 3. Eijkman Research Center for Molecular Biology, BRIN, Indonesia; 4. Exeins Health Initiative, Jakarta, Indonesia; 5. Infectious Diseases Society Kota Kinabalu Sabah-Menzies School of Health Research Clinical Research Unit, Kota Kinabalu, Sabah, Malaysia; 6. Clinical Research Centre-Queen Elizabeth Hospital, Ministry of Health, Kota Kinabalu, Sabah, Malaysia; 7. School of Medicine and Health Sciences, Monash University Malaysia, Kuala Lumpur, Malaysia; 8. Centre for Tropical Bioinformatics and Molecular Biology, James Cook University, Cairns, Queensland, Australia; 9. Biotechnology Research Institute, Universiti Malaysia Sabah, Kota Kinabalu, Sabah, Malaysia.; 10. Faculty of Science and Natural Resources, Universiti Malaysia Sabah, Kota Kinabalu, Sabah Malaysia.; 11. Faculty of Medicine and Health Sciences, Universiti Malaysia Sabah, Kota Kinabalu, Sabah, Malaysia; 12. Monash University Indonesia, Tangerang, Indonesia; 13. Oxford University Clinical Research Unit Indonesia, Faculty of Medicine Universitas Indonesia, Jakarta, Indonesia; 14. U.S. Naval Medical Research Unit INDO PACIFIC, Singapore; 15. Drug Resistance and Diagnostics, Australian Defence Force Malaria and Infectious Disease Institute, Brisbane, Queensland, Australia; 16. Malaysian Armed Forces and Faculty of Medicine & Defence Health, National Defence University of Malaysia; 17. QIMR Berghofer Medical Research Institute, Brisbane, Queensland, Australia; 18. Department of Infection Biology, London School of Hygiene & Tropical Medicine, London, United Kingdom

**Keywords:** zoonotic malaria, *Plasmodium*, *P. knowlesi*, *P. cynomolgi*, *P. vivax*, PCR, reverse transcription, limit of detection, dried blood spots

## Abstract

**Background:**

Zoonotic *P. knowlesi* and *P. cynomolgi* symptomatic and asymptomatic infections occur across endemic areas of Southeast Asia. Most infections are low-parasitemia, with an unknown proportion below routine microscopy detection thresholds. Molecular surveillance tools optimizing the limit of detection (LOD) would allow more accurate estimates of zoonotic malaria prevalence.

**Methods:**

An established ultra-sensitive *Plasmodium* genus quantitative-PCR (qPCR) assay targeting the 18S rRNA gene underwent LOD evaluation with and without reverse transcription (RT) for *P. knowlesi*, *P. cynomolgi* and *P. vivax* using total nucleic acid preserved (DNA/RNA Shield^™^) isolates and archived dried blood spots (DBS). LODs for selected *P. knowlesi-*specific assays, and reference *P. vivax-* and *P. cynomolgi*-specific assays were determined with RT. Assay specificities were assessed using clinical malaria samples and malaria-negative controls.

**Results:**

The use of reverse transcription improved *Plasmodium* species detection by up to 10,000-fold (*Plasmodium* genus), 2759-fold (*P. knowlesi*), 1000-fold (*P. vivax*) and 10-fold (*P. cynomolgi*). The median LOD with RT for the Kamau et al. *Plasmodium* genus RT-qPCR assay was ≤0.0002 parasites/μL for *P. knowlesi* and 0.002 parasites/μL for both *P. cynomolgi* and *P. vivax*. The LODs with RT for *P. knowlesi*-specific PCRs were: Imwong et al. 18S rRNA (0.0007 parasites/μL); Divis et al. real-time 18S rRNA (0.0002 parasites/μL); Lubis et al. hemi-nested *SICAvar* (1.1 parasites/μL) and Lee et al. nested 18S rRNA (11 parasites/μL). The LOD for *P. vivax-* and *P. cynomolgi-*specific assays with RT were 0.02 and 0.20 parasites/μL respectively. For DBS *P. knowlesi* samples the median LOD for the *Plasmodium* genus qPCR with RT was 0.08, and without RT was 19.89 parasites/uL (249-fold change); no LOD improvement was demonstrated in DBS archived beyond 6 years. The *Plasmodium* genus and *P. knowlesi*-assays were 100% specific for *Plasmodium* species and *P. knowlesi* detection, respectively, from 190 clinical infections and 48 healthy controls. Reference *P. vivax-*specific primers demonstrated known cross-reactivity with *P. cynomolgi*.

**Conclusion:**

Our findings support the use of an 18S rRNA *Plasmodium* genus qPCR and species-specific nested PCR protocol with RT for highly-sensitive surveillance of zoonotic and human *Plasmodium* species infections.

## Author Summary

The monkey malaria parasite *Plasmodium knowlesi* has been found to increasingly infect humans across Southeast Asia via the bite of it’s anopheline mosquito vectors. Human infections with a similar monkey parasite, *Plasmodium cynomologi,* have also been reported. The diagnostic tools commonly used to detect these malaria species are often unable to detect very low-level infections. We aimed to to improve surveillance detection tools and blood sample collection methods to detect these zoonotic malaria species and understand the extent of transmission and the burden of disease. This study validated and compared the use of molecular laboratory assays targeting these species. We found that with the use of reverse transcription, large improvements in the limit of detection were possible, by up to 10,000-fold for initial malaria screening, and up to 2759-fold for specific *P. knowlesi* detection. Findings from this study support the use of ultrasensitive detection tools to improve surveillance approaches to emerging zoonotic malaria species.

## BACKGROUND

*Plasmodium knowlesi* is a unicellular protozoan malaria parasite present across Southeast Asia within the geographical range of its natural monkey hosts and vector mosquitoes (1,2). *P. knowlesi* is the most common cause of human malaria in Malaysia; capable of causing severe disease comparable to *P. falciparum* (3-5). Human infections with other genetically similar zoonotic species, such as *P. cynomolgi* which share the same natural macaque hosts, have also been reported (6). Accurate detection of emerging zoonotic species such as *P. knowlesi* and *P. cynomolgi* in co-endemic areas with other human *Plasmodium* species infections is necessary to understand the geographical extent of zoonotic malaria transmission and to improve regional estimates of the disease burden (7). Improving national malaria control program detection and reporting of low-level *P. knowlesi* infections is also vital to demonstrate World Health Organization (WHO)-certified elimination for other non-zoonotic malaria species in Southeast Asia (8).

Conventional malaria diagnostic methods such as microscopy lack sensitivity and specificity for active surveillance of *P. knowlesi* due to common low-level sub-microscopic infections and an inability to accurately distinguish other morphologically similar *Plasmodium* species (7,9); notably *P. malariae* and the early ring stages of *P. falciparum* (10,11). Similarly, *P. cynomolgi* microscopically resembles *P. vivax* in human infections (12,13). Current malaria rapid diagnostic tests which detect circulating *Plasmodium* species antigens also remain insufficiently sensitive for *P. knowlesi* passive case detection at the low parasite counts able to produce symptomatic infections (14-16). Multiple molecular methods to detect *P. knowlesi* have been developed, including both quantitative and conventional qualitative PCR assays (7,17). However, systematic comparisons of the lower limit of detection (LOD) of these assays and exhaustive testing of *Plasmodium* species-specificity are currently lacking (7). The degree to which the LOD is enhanced with a prior reverse transcription (RT) step is not well characterised despite potential benefits in improving the detection of very low-level parasitemia symptomatic or asymptomatic zoonotic *Plasmodium* species infections (18). Specificity for *P. knowlesi* detection also ideally needs to be validated against other macaque zoonotic *Plasmodium* species, including *P. cynomolgi* (7).

To support an improved molecular surveillance workflow for detection of low-level zoonotic *Plasmodium* species infections (7,19), we evaluated an established *Plasmodium* genus and species-specific PCR assays with the inclusion of a reverse transcription step to enhance the LOD in both total nucleic acid preserved media and dried blood spot (DBS) collected samples.

## METHODS

### Clinical specimen collection and storage

Clinical venous whole blood samples and dried blood spots were collected prior to antimalarial treatment from individuals diagnosed with malaria by routine hospital microscopy, as part of an ongoing prospective malaria study in Sabah, Malaysia between April 2013 and May 2023. Adult healthy individuals were recruited as malaria-negative controls. Additional *P. vivax* clinical cases were enrolled from a prospective malaria study in western Indonesia between Jan 2022 and Aug 2023. Whole blood samples were collected in ethylenediaminetetraacetic acid (EDTA) and a small subset in DNA/RNA Shield^™^ (Zymo Research, Irvine, CA, USA) before being frozen at −80°C at the time of enrolment. DBS were concurrently made using 20μL whole blood spotted on Whatman 3M filter paper and then stored in sealed bags with desiccant. A single donated *P. cynomolgi*-infected sample (20) obtained from a macaque host was frozen in glycerolyte and stored in liquid nitrogen prior to thawing, counting and immediately placing in DNA/RNA Shield^™^.

### Microscopic parasite count quantification

Microscopic diagnosis of *Plasmodium* species was undertaken by experienced research microscopists using thick and thin Giemsa-stained blood films. Microscopic quantification of parasitemia (parasites per microlitre) was performed using thick blood smears calculated from the number of parasites per 200 white blood cells, multiplied by the individual patient’s total white cell count obtained from routine hospital laboratory flow cytometry (21).

### Total nucleic acid extraction and PCR amplification

Total nucleic acids were directly extracted from 200 μL of whole blood using a QIAamp^®^ DNA Blood Mini Kit (QIAGEN, Cat. No. 51106), with DNA/RNA Shield^™^ samples eluted in 50ul AE buffer to account for the preservative dilution factor. DNA and RNA extraction from DBS were carried out using an in-house method, established by Zainabadi *et al*, 2017 (22). Briefly, DBS equivalent to 40μl whole blood was incubated with 900μl lysis buffer at 65°C, with shaking at 250rpm for 90 minutes. Lysate was transferred to QIAamp^®^ DNA Blood Mini Kit (QIAGEN, Cat. No. 51106) columns, washed with modified buffers, dried at 65°C for 10 minutes and eluted in 40μl buffer AE. The primers, annealing temperatures and/or probe sequences for each PCR assay are described in Table 1. Real-time PCR amplifications were performed on a QuantStudio^™^ 6 Flex (Applied Biosystems). Conventional PCR was performed on a DNA thermal cycler (Bio-Rad T100^™^ thermal cycler). The amplified nested PCR products were separated by electrophoresis using 2% agarose gels, stained by SYBR Safe^™^ (Invitrogen), and visualised on a UV transilluminator (Gel Doc XR+ imaging system, Bio-Rad). Each PCR amplification included a *Plasmodium* species positive and negative control and molecular weight standards (Applied Biosystems^™^).

### *Plasmodium* species confirmation using reference PCR

A validated reference PCR targeting the *Plasmodium* 18S rRNA genes was used to confirm the *Plasmodium* species using EDTA-whole blood clinical samples, consisting of an initial *Plasmodium* genus (hereafter abbreviated to *P*. genus) nest 1, followed by species-specific nest 2 for *P. falciparum*, *P. vivax*, *P. malariae*, and *P. ovale* spp. (23). The *P. knowlesi*- (24) and *P. cynomolgi*-specific (25) reference assays used also target 18S rRNA genes. The *P. knowlesi* reference assay has a previously reported high sensitivity (LOD less than 10 parasite genomes) when validated without reverse transcription (RT) against four *P. knowlesi* strains (Malayan, H, Philippine, and Hackeri); specificity was assessed against the major human *Plasmodium* species in addition to other simian *Plasmodium* species present in Southeast Asia including *P. cynomolgi*, *P. inui*, and *P. simiovale* (24).

### Selection of PCR assays for LOD evaluation

**Figure 1.**
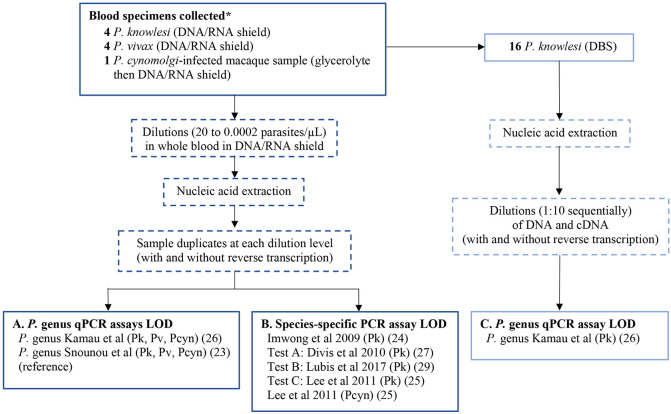
Limit of detection workflow for (A) *P.* genus, (B) *Plasmodium* species-specific, and (C) *P.* genus dried blood spot PCR assays Abbreviations: DBS = dried blood spot; LOD = limit of detection; qPCR = real-time quantitative PCR; Pk = *P. knowlesi;* Pf = *P. falciparum,* Pv = *P. vivax;* Pcyn = *P. cynomolgi;* RT = reverse transcription

A reverse transcriptase real-time hydrolysis probe (RT-qPCR) assay designed to detect the *P. lasmodium* genus was evaluated for its utility in enhancing LOD (Figure 1A) (26). Three published *P. knowlesi* species-specific assays, which have not previously had their LODs evaluated using reverse transcription, were compared to the reference assay (Figure 1B). Test A is a qPCR assay (27) in current use in the State Public Health Laboratory (Makmal Kesihatan Awam; MKA) Sabah, Malaysia, for routine *P. knowlesi* malaria detection (28). Test B is a hemi-nested PCR targeting the multicopy *SICAvar* genes, previously validated for *P. knowlesi* detection against *P. falciparum*, *P. vivax*, *P. malariae*, and *P. ovale* spp., in addition to clinical *P. knowlesi* isolates from Sarawak, Malaysia (29). Test C is a nested conventional PCR assay targeting 18S rRNA genes specific for *P. knowlesi* that has been robustly validated against *P. vivax* and other zoonotic *Plasmodium* species (25).

### Limit of detection evaluation for PCR assays with and without RT

The initial microscopy-quantified *Plasmodium* species infected samples collected in DNA/RNA Shield^™^ were diluted with malaria-negative whole blood (also at the same manufacturer recommended DNA/RNA Shield^™^ ratio) to prepare individual parasite count dilutions ranging between 20 to 0.0002 parasites/μL. Total nucleic acids from samples at each serial parasite count dilution were then extracted and duplicate aliquots prepared. High-capacity cDNA reverse transcription (Applied Biosystems^™^, Thermo Fisher Scientific, MA, USA) was then performed on one aliquot from each pair. PCR was performed on the paired aliquots to detect *P.* genus by the qPCR assay (26), followed by species-specific assays for: *P. knowlesi* by the reference hemi-nested assay (24) and test assays A, B , C (25,27,29); *P. vivax* (23) and *P. cynomolgi* (25) by reference assays, in addition to the newly designed *P. vivax* primers. The LOD was expressed as the lowest parasite count per microlitre of whole blood detected by an individual PCR assay in both amplification replicates (Figure 1C).

### Limit of detection of *P.* genus qPCR assay for dried blood spots (DBS) with and without RT

The LOD for the *P.* genus qPCR assay was also evaluated using archived DBS from *P. knowlesi* clinical infections with and without RT (Figure 1C). DBS were stored individually after collection with dessicant at room temperature unexposed to light before processing. Nucleic acids were extracted from the DBS samples, with 10μl immediately reverse transcribed into cDNA. *P.* genus qPCR was conducted on serial 1:10 DNA and cDNA dilutions with the LOD calculated using the initial enumerated parasitemia divided by the corresponding dilution level.

### Evaluation of PCR assay specificity on clinical malaria samples

For the analysis of *P. knowlesi*, *P. falciparum*, and *P. vivax* clinical isolates, in addition to a *P. cynomolgi* macaque-derived isolate, all samples were individually tested using the *P.* genus qPCR assay and the three *P. knowlesi*-specific PCR assays. Results were compared against the reference PCR. Specificity for the *P.* genus assay was evaluated for malaria detection (any *Plasmodium* species) versus malaria-negative controls, and for species-specific assays using the corresponding *Plasmodium* species infection versus other *Plasmodium* species and malaria-negative controls combined. Clinical blood samples collected in EDTA likely had RNA degradation upon thawing; therefore, reverse transcription was not performed for this part of the analysis.

### Statistical analyses

Parasite counts for each clinical *Plasmodium* species infection were summarised using median and interquartile range (IQR). The median whole blood LOD was calculated with and without reverse transcription for the *P. knowlesi* and *P. vivax* isolates. To calculate the LOD fold-change, the LOD without RT was divided by the LOD with RT. One-way ANOVA was used to test for differences in parasite count distribution across *Plasmodium* species, followed by Student’s t-test for pairwise comparisons of log-transformed data. Results of PCR assays evaluated against reference PCR were defined as true positive (TP), false negative (FN), true negative (TN), and false positive (FP), enabling calculation of diagnostic sensitivity (TP/TP+FN) and specificity (TN/TN+FP) with exact binomial 95% confidence intervals. All statistical analyses were performed using Stata version 17.0 (StataCorp, Texas, USA).

## RESULTS

### Limit of detection of *P.* genus PCR assays

**Figure 2.**
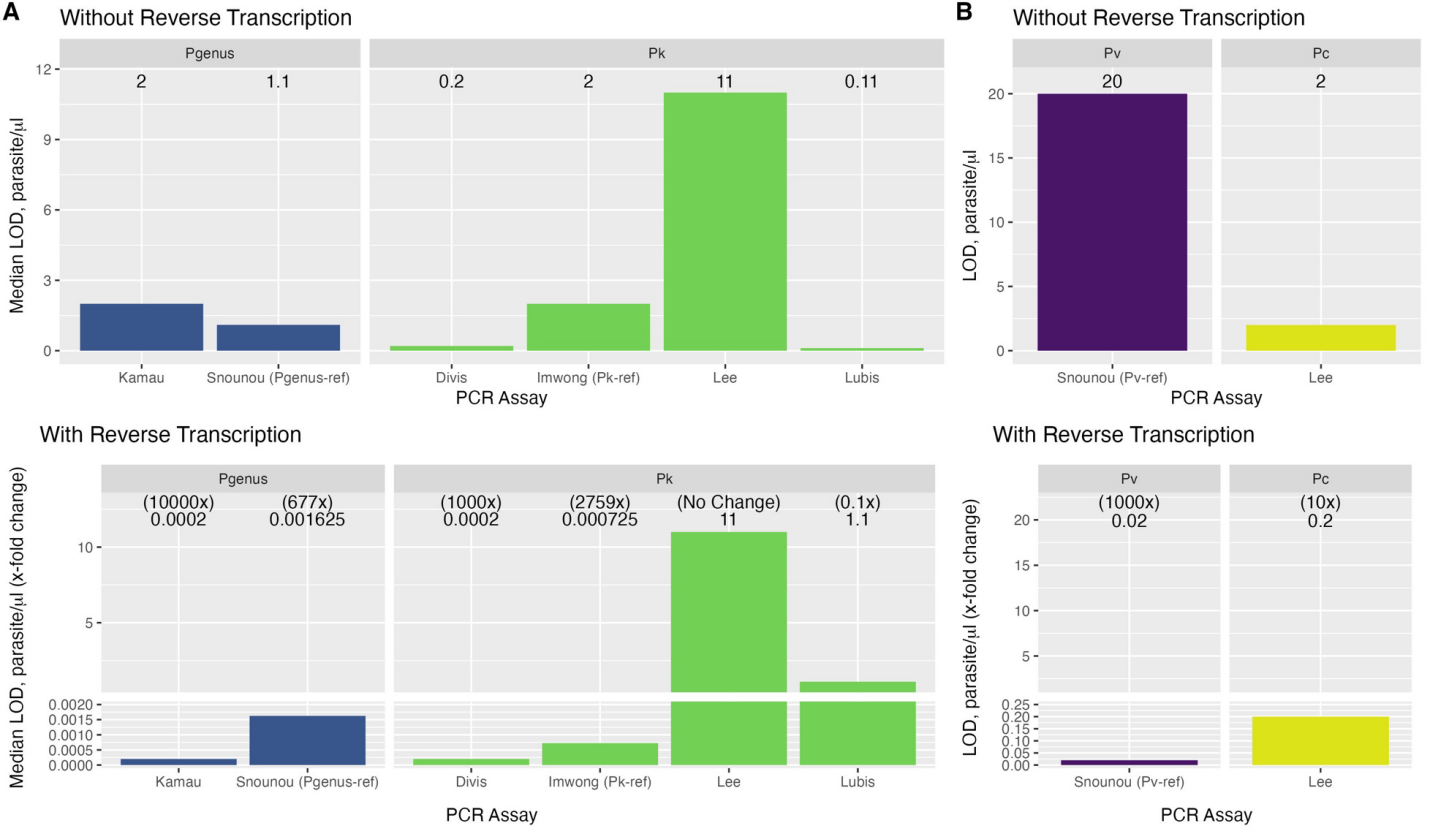
The median LOD and fold-change with and without reverse transcription for PCR assays to detect *Plasmodium* species. **(A)** The median LOD (parasites/μL) for *P.* genus assays using 6 clinical isolates (*P. knowlesi*=4, *P. vivax*=1, *P. cynomolgi*=1) and *P. knowlesi*-specific assays using 4 clinical isolates; **(B)** The LOD (parasites/μL) for *P. vivax* (n=1) and *P. cynomolgi* (n=1) specific assays. Abbreviations: Pgenus, *Plasmodium* genus; Pk, *P. knowlesi*; Pv, *P. vivax;* Pc, *P. cynomolgi*

The LOD was performed on *P. knowlesi* (n=4), *P. vivax* (n=4) and *P. cynomolgi* (n=1) whole blood isolates. For the *P.* genus Kamau et al. qPCR assay, without reverse transcription, the median LOD to detect each individual *Plasmodium* species was 2 parasites/μL (Table 2 and Figure 2A). With reverse transcription, the assay sensitivity for *P. knowlesi* improved with a LOD of ≤0.0002 parasites/μL for all four isolates (10,000-fold change). The LOD for both *P. vivax* and *P. cynomolgi* improved to 0.002 parasites/μL with RT (1,000-fold change); Figure 2B. In comparison, the reference Snounu *P.* genus assay had a slightly higher LOD for *P. vivax* and *P. cynomolgi* without RT (0.2 parasites/μL for both); however, with RT a less pronounced improvement in LOD was demonstrated at 0.02 and 0.01 parasites/μL, respectively.

### Limit of detection of *Plasmodium* species*-*specific PCR assays

For the *P. knowlesi*-specific assays, the median LOD without and with reverse transcription was 2 and 0.0007 parasites/μL, respectively, for the Imwong et al. reference assay (2759-fold change); 0.2 and 0.0002 parasites/μL, respectively, for Test A (1000-fold); 0.11 and 1.1 parasites/μL, respectively, for Test B (no improvement); and 11 parasites/μL for both (no change) for Test C (Table 2 and Figure 2A).

Without reverse transcription, the LODs using species-specific reference assays were 2 parasites/μL for *P. cynomolgi* and 20 parasites/μL for *P. vivax*. With reverse transcription, the LODs were 0.2 (10-fold change) and 0.02 (1000-fold change) for *P. cynomolgi* and *P. vivax* respectively. However, additional testing of the *P. cynomolgi* isolate using the reference *P. vivax*-targeted rVIV1/rVIV2 primers (23) amplified this target from the macaque-origin *P. cynomolgi* infection both without (0.2 parasites/μL) and with reverse transcription (0.02 parasites/μL), producing a false-positive *P. vivax* result (Table 2 and Figure 2B).

The LOD with reverse transcription of the *P.* genus assay was comparable to the LOD of the best performing species-specific assay for *P. knowlesi* detection (≤0.0002 parasites/μL for both). In contrast, the *P.* genus assay had a superior LOD compared to the reference species-specific assays for *P. vivax* (0.002 vs 0.02 parasites/μL respectively) and *P. cynomolgi* (0.002 vs 0.2 parasites/μL respectively).

### Limit of detection of *P.* genus qPCR for dried blood spots (DBS)

The median LOD between DNA and cDNA generated from dried blood spots (DBS) for 4 *P. knowlesi* extracted samples collected 8 months prior to evaluation of the *P.* genus Kamau 2011 assay without and with RT was 19.86 and 0.08 parasites/uL, respectively (249-fold change); Table 3. Archived DBS samples (n=12) collected more than 6 years (up to 11 years) prior to extraction demonstrated a similar LOD with and without RT (median 20 parasites/μL).

### Specificity of *P.* genus and individual *Plasmodium* species PCR assays

A total of 239 samples were included in the clinical evaluation of test specificity without RT, consisting of 96 *P. knowlesi*, 50 *P. vivax,* 44 *P. falciparum*, and 1 *P. cynomolgi* infected samples, and 48 malaria-negative controls. The median parasitemia was 1,957/μL (IQR 261-5,762; range 27-210,100 parasites/μL) for *P. knowlesi*, 3,246/μL (IQR 1,588-7,306; range 77-20,064 parasites/μL) for *P. vivax*, and 14,015/μL (IQR 2,193-33,413; range 34-297,000 parasites/μL) for *P. falciparum*.

The *P.* genus qPCR screening assay was both 100% specific and sensitive for the detection of *Plasmodium* species overall compared to the reference PCR, with all 48 samples from uninfected controls confirmed as malaria-negative.

*P. knowlesi*- assays for Tests A (27) and B (29) correctly identified all 96 *P. knowlesi* and 95 non-*P. knowlesi* samples, resulting in 100% specificity and sensitivity. Test C (25) was negative for a single *P. knowlesi* isolate with a parasite count of 1,535 parasites/μL, resulting in 99% (95% CI 94.3-100.0) sensitivity and 100% specificity. The reference assays for *P. falciparum* and *P. vivax* (23), and for *P. cynomolgi* (25) were negative for all *P. knowlesi* clinical isolates tested (Table 2).

## DISCUSSION

Malaria-susceptible countries in most of Southeast Asia, including those approaching or achieving WHO elimination of major human-only *Plasmodium* species, remain at-risk for zoonotic malaria transmission (30,31). Understanding regional heterogeneity in *P. knowlesi* transmission intensity and disease morbidity will require the selective deployment of highly sensitive and specific molecular detection tools for both diagnostic and surveillance purposes (19). Our major finding demonstrated that the use of a reverse-transcription step after extraction of preserved total nucleic acids in clinical samples considerably improves the limit of detection of both the selected *P.* genus RT-qPCR screening assay, and *P. knowlesi*-specific assays (reference and Test A; both >1000-fold) by additionally amplifying ribosomal RNA sequences. The enhanced limit of detection was consistent across both field-stable DNA/RNA Shield^™^ samples and to a lesser degree in recent (although not older) dried blood spots. The second key finding of this study was the excellent performance of the *P.* genus screening assay, originally developed and validated for use in an African context for *P. falciparum* (26), to detect previously unvalidated species including *P. knowlesi*, *P. cynomolgi* and *P. vivax*. The specificity of each of the *P. knowlesi*-targeted assays to exclude *P. cynomolgi* and non-zoonotic *Plasmodium* species was confirmed to be excellent. Together these findings highlight the potential utility of incorporating these assays in a molecular surveillance approach to detect both human and zoonotic *Plasmodium* species that are well below the reported parasite count detection limits for current conventional PCR, loop-mediated isothermal amplification, microscopy or parasite lactate-dehydrogenase-based rapid diagnostic tests (19).

The community-based detection of submicroscopic *P. knowlesi* and *P. cynomolgi* infections, both symptomatic or asymptomatic, requires ultrasensitive molecular tools to understand the true extent of population-level transmission (19). Recent studies in areas of both Peninsular Malaysia and the East Malaysian state of Sarawak have reported human infections in local communities living in or near forested areas with other macaque malaria species, including *P. inui*, *P. coatneyi, P. fieldi* and possibly *P. simiovale,* in addition to *P. knowlesi* and *P. cynomolgi* (32). It is unclear whether or to what extent these low-level zoonotic infections may facilitate onward transmission to humans (33,34), as occurs with low parasitemia *P. falciparum* and *P. vivax* infections (35,36), although sustained human-to-human transmission of *P. knowlesi* has not been evident to date(25,37).

The selection of the major RT-qPCR *P.* genus screening assay aimed to maximise the detection limits for low-level zoonotic *Plasmodium* species infections due to the known high multicopy number (5 to 10 copies per genome depending on the *Plasmodium* species) of the 18S rRNA target (38), in addition to amplification of both the A- and S- type genes and their RNA transcripts (26). The excellent LOD demonstrated with the *P.* genus RT-qPCR assay in the current study of <0.0002 parasites/μL for *P. knowlesi* detection is consistent with a previously reported extremely low LOD of ~0.0004 parasites/μL for clinical *P. falciparum* samples (26). DNA-concentrated packed red blood cell samples have been demonstrated to further improve sensitivity for *P. falciparum* detection in population-based malaria prevalence surveys in elimination areas of Thailand (39). Comparable performance to this *P.* genus RT-qPCR assay was reported with a separately established ultrasensitive quantitative PCR method (uPCR) with a limit of detection of 0.022 parasites/μL (39), however, a major advantage of the reverse transcriptase qPCR assay is the requirement for comparatively lower blood volumes (18). Interestingly, in the current study the conventional PCR *P.* genus reference assay also demonstrated a large increase in analytical sensitivity after reverse transcription (~1800-fold) and may provide a more cost-effective option compared to qPCR for surveillance purposes. DNA/RNA Shield^™^ was selected as the preferred blood collection preservation method over other media due to its reported ability to stabilise DNA/RNA at ambient temperatures in field settings and compatibility with most DNA and RNA purification kits for subsequent high-throughput workflows including reverse transcription (40).

To date, only a few studies have incorporated reverse transcription in the molecular detection of *Plasmodium* species (41,42). The reverse transcription step in the present study improved the analytical sensitivity of our selected assays to detect zoonotic *P. knowlesi*, *P. cynomolgi* and other human malaria infections by up to 10,000-fold (*Plasmodium* genus), 2759-fold (*P. knowlesi*), 1000-fold (*P. vivax*) and 10-fold (*P. cynomolgi*), respectively. The *P. knowlesi*-specific hemi-nested reference assay with reverse transcription demonstrated a comparable limit of detection to the qPCR Test A (which requires an expensive real-time hydrolysis probe), and was superior to both Test B targeting *SICavar* and Test C targeting the 18S rRNA gene. Without reverse transcription, the lowest limit of detection for *P. knowlesi* was seen with Test B, suggesting that the multiple chromosomal copies of the variant antigen *SICAvar* provide equivalent or better signal amplification than the detection of transcripts from whichever of these gene copies is activated in any particular parasite cell in the peripheral blood. Constraints on the widespread use of reverse transcription include the additional cost, laboratory time, and the usual rapid degradation of RNA molecules in field or laboratory settings. However, the degree of RNA amplification with reverse transcription was aided in our study by collecting blood samples in room-temperature stable RNA preservation media suitable for field-based surveillance, which also allows other potential downstream pathophysiological or transcriptomic analyses dependent on pathogen or host RNA transcripts.

The low reported limit of detection for the RT-qPCR *P.* genus assay conducted on DBS in this study (~0.08 parasites/μL with reverse transcription) suggests this type of sample collection would also enable the identification of a large proportion of submicroscopic and/or asymptomatic infections. DBS collection is logistically a more feasible, inexpensive and acceptable option particularly for asymptomatic or younger participants (given the need for fingerprick blood collection rather than venepuncture) for large-scale malaria surveillance surveys. However, the reverse transcription step only improved the LOD in DBS samples that were collected within 8 months; older DBS stored in recommended conditions for more than 6 years did not provide any improvement in the LOD with and without RT due to likely degradation of RNA. Regardless, the *P.* genus LOD of around 2 to 20 parasites/μL without RT for DBS samples remains encouraging as a first-line option for surveillance screening purposes, although the use of DBS would require further evaluation with *Plasmodium* species-specific PCR assay differentiation.

The current study confirmed previous findings detailing cross-reactivity between the nested PCR primers for *P. vivax* (rVIV1/rVIV2) with *P. cynomolgi* (13). A single mismatch in the 30 nucleotides of the rVIV2 primer sequences was reported to cross-amplify *P. cynomolgi* isolates (13). The nested *P. vivax-*specific assay using the same primers rVIV1/rVIV2 designed to target the 18S rRNA gene also amplified *P. cynomolgi* in our LOD analysis (23). The separate *P. cynomolgi* primers remained highly specific and did not erroneously amplify *P. vivax* or other closely related *Plasmodium* species DNA. In practice, the concurrent use of both assays would enable accurate identification of a *P. vivax* mono-infection, however, this approach would not be able to differentiate a *P. vivax/P. cynomolgi* co-infection from a *P. cynomolgi* mono-infection. Mis-identification of symptomatic *P. cynomolgi* infections as *P. vivax* would not result in inappropriate treatment, given both have a latent hypnozoite liver life-stage requiring additional radical cure with primaquine. Most other commonly used single-round multiplex (43) and qPCR assays (44) containing *P. vivax-*specific targets have also not been validated against isolates of *P. cynomolgi* or other closely related macaque *Plasmodium* species. However, a variety of sequencing approaches of targeted gene amplicons including mitochondrial COX1 and cytochrome b, *SICAvar* and SSU 18S rRNA followed by sequencing and reference alignment have been used to confirm unknown or mixed zoonotic *Plasmodium* species infections following initial ambiguous PCR results (45,46).

A limitation of this study was the inability to validate submicroscopic clinical *P. knowlesi* infections and other zoonotic species such as *P. fieldi*, *P. inui* and *P. coatneyi* for which samples were not available. Due to the increasing number of published *P. knowlesi* assays, we were not able to evaluate other *P. knowlesi* assays of possibly comparable performance within our selected workflow. We were also unable to evaluate mixed infections of *P. knowlesi*, *P. vivax* and *P. falciparum* despite these cases being reported in certain areas such as Indonesia (29) and Vietnam (47). Due to sample availability, we were also restricted to only a single *P. cynomolgi* sample to determine the LOD of the *P. cynomolgi-*specific assay (25). The discrepancy between the LOD for the *P.* genus qPCR screening assay and the species-specific assay for *P. cynomolgi* detection may mean a proportion of very low-level *P. cynomolgi* infections are unable to be identified beyond a *P.* genus threshold using the current protocol.

## CONCLUSIONS

The *Plasmodium* genus reverse transcriptase qPCR assay can provide highly sensitive screening for zoonotic and human malaria, including for submicroscopic infections in at-risk populations in endemic areas. The use of this molecular surveillance protocol for either whole blood or DBS collected samples in understudied areas of Southeast Asia would enable improved understanding of the regional disease burden and transmission dynamics of zoonotic malaria. Enhanced molecular tools and future iterative improvements to conventional surveillance protocols are especially critical as Southeast Asia continues to exert considerable public health efforts towards human malaria elimination despite the challenge of additional zoonotic *Plasmodium* species infections at an expanding human-animal-interface.

## Figures and Tables

**Table 1.** Primer sequences and annealing temperatures of *Plasmodium* genus and species-specific PCR assays

Assay ID	PCR assays	Primer / Probe	Sequence (5’ – 3’)	Annealing temp. (°C)	PCR method	*Plasmodium* gene target
*Plasmodium* spp. screening	Kamau et al, 2011 (26) (rRNA template)	KamGF KamGR KamGP probe	GCTCTTTCTTGATTTCTTGGATG AGCAGGTTAAGATCTCGTTCG ^FAM^-ATGGCCGTTTTTAGTTCGTG-^BHQ1^	60	RT-qPCR	18S rRNA
Test A (Pk)	Divis et al, 2010 (27)	Plasmo 1 Plasmo 2 Pk probe	GTTAAGGGAGTGAAGACGATCAGA AACCCAAAGACTTTGATTTCTCATAA ^FAM^-CTCTCCGGAGATTAGAACTCTTAGATTGCT-^BHQ1^	60	qPCR	18S rRNA
Test B (Pk)	Lubis et al, 2017 (29)	SICAf1 SICAr1 SICAf2 SICAr1	GGTCCTCTTGGTAAAGGAGG CCCTTTTTGACATTCGTCC CTTGGTAAAGGAGGACCACG CCCTTTTTGACATTCGTCC	55	Hemi-nested	SICAvar
Test C (Pk)	Lee et al, 2011 (25)	rPLU 1 rPLU 5 Kn1f Kn3r	TCAAAGATTAAGCCATGCAAGTGA CCTGTTGTTGCCTTAAACTTC CTCAACACGGGAAAACTCACTAGTTTA GTATTATTAGGTACAAGGTAGCAGTATGC	66	Nested	18S rRNA
Test D (Pcyn)	Lee et al, 2011 (25)	CY2F CY4R	GATTTGCTAAATTGCGGTCG CGGTATGATAAGCCAGGGAAGT	66		
Pk reference	Imwong et al, 2009 (24)	PkF1160 PkF1150 PkF1140 PkR1150	GATGCCTCCGCGTATCGAC GAGTTCTAATCTCCGGAGAGAAAAGA GATTCATCTATTAAAAATTTGCTTC GAGTTCTAATCTCCGGAGAGAAAAGA	55 50	Hemi-nested	18S rRNA
Human-species reference	Snounou et al. (1993) (23)	rPLU6 rPLU5 rFAL 1 rFAL 2 rVIV 1 rVIV 2 rMAL 1 rMAL 2 rOVA 1 rOVA 2	TTAAAATTGTTGCAGTTAAAACG CCTGTTGTTGCCTTAAACTTC TTAAACTGGTTTGGGAAAACCAAATATATT ACACAATGAACTCAATCATGACTACCCGTC CGCTTCTAGCTTAATCCACATAACTGATAC ACTTCCAAGCCGAAGCAAAGAAAGTCCTTA ATAACATAGTTGTACGTTAAGAATAACCGC AAAATTCCCATGCATAAAAAATTATACAAA ATCTCTTTTGCTATTTTTTAGTATTGGAGA GGAAAAGGACACATTAATTGTATCCTAGTG	58	Nested	18S rRNA

**Table 2.** Limit of detection (LOD) of *Plasmodium* genus and species-specific PCR assays

PCR assay	Target *P.* genus/species	Tested species	LOD without RT	LOD with RT	Fold change post RT (x)
Kamau et al. (26)	*P.* genus	Pk	2	≤0.0002	10000
Kamau et al. (26)	*P.* genus	Pv	2	0.002	1000
Kamau et al. (26)	*P.* genus	Pc	2	0.002	1000
Snounou et al. (23)	*P.* genus	Pk	2	0.0011	1818
Snounou et al. (23)	*P.* genus	Pv	0.2	0.02	10
Snounou et al. (23)	*P.* genus	Pc	0.2	0.01	20
Imwong et al. (24)	Pk	Pk	2	0.0007	2759
Divis et al. (27)	Pk	Pk	0.2	≤0.0002	1000
Lee et al. (25)	Pk	Pk	11	11	No change
Lubis et al. (29)	Pk	Pk	0.11	1.1	0.1*
Snounou et al. (23)	Pv	Pv	20	0.02	1000
Snounou et al. (23)	Pv	^#^Pc	0.2	0.02	10
Lee et al. (25)	Pc	Pc	2	0.2	10

Abbreviations: *P.* genus = *Plasmodium* genus; Pk = *P. knowlesi*; Pv = *P. vivax*; Pc = *P. cynomolgi*; RT = reverse transcription

LOD is the lowest parasitemia (parasites/μL) detected by all three replicates for each PCR assay

LOD and fold-change post RT are reported as median for *P. knowlesi* (n=4)

* There was no improvement in LOD by Lubis PCR assay (Test B) after RT

# *P. vivax* assay cross-reacts with *P. cynomolgi*

**Table 3.** Limit of detection of the 18S rRNA *P.* genus qPCR assay for *P. knowlesi* dried blood spot samples

Species evaluated	Initial parasitemia (/μL)	Time from DBS collection	LOD without RT (DNA)	LOD with RT (cDNA)	Fold change post RT (x)
**Recent samples**					
Pk	2663	8 months	26.6	0.027	1000
Pk	1377	8 months	13.8	0.138	100
Pk	198	8 months	2.0	0.020	100
Pk	26	8 months	26.0	2.600	10
*Median (IQR)*	788 (198-1377)	8 months	19.9 (13.8-26.0)	0.08 (0.03-0.14)	100 (100-100)
**Archived samples** ^†^					
Pk *(N=12)*	372 (177-2604)	11 years (8-11)*	20 (2.5-20)	20 (2-20)	1 (1-1.25)

Limit of detection (parasites/μL) with and without RT of each *P. knowlesi* isolate was conducted in duplicate for each target species at each dilution level.

† Results are median (IQR)

* Range from 6 to 11 years

Pk = *P. knowlesi*
